# Preserving Cultural Diversity in Rural Africa Using Renewable Energy

**DOI:** 10.1002/gch2.202300263

**Published:** 2023-11-27

**Authors:** Emil Roduner, Egmont R. Rohwer

**Affiliations:** ^1^ Chemistry Department University of Pretoria Pretoria 0002 South Africa; ^2^ Institute of Physical Chemistry University of Stuttgart 70569 Stuttgart Germany

**Keywords:** cultural diversity, DC mini‐grids, future Africa, improved living conditions, renewable energy, smart villages

## Abstract

Ninety percent of the large interior, rural part of Africa is not an abundant user of fossil fuels and is not connected to an electricity grid. This limits habitability and leads to significant migration to larger cities in attempts to improve economic and social welfare, which happens at the cost of its rich cultural diversity by inevitable adaption and mixing of societies. A direct transition from a firewood to an off‐grid renewable electricity age can mitigate this detrimental development. This perspective discusses the interdisciplinary requirements linking cultural, sociological, economic, and technical aspects for a transition to modern life without loss of valuable traditions. Photovoltaic power and wind energy can provide local affordable electricity in off‐grid locations. Intermediate storage for day–night cycles is catered for by novel types of batteries. Purifying and recycling water, refrigerating food and medicine, and benefitting from contact with the world via electronic media permit a tremendous increase in living conditions and significantly lower the pressure of migration into cities. Access to energy is a fundamental requirement for the preservation of the rich cultural diversity with family and tribal bindings, local languages, traditions, and religions, and allows for a more moderate transition to a modern society.

## Graphical abstract

Renewable energy can provide the missing electricity in the large off‐grid part of Sub‐Saharan Africa. This leads to tremendously improved habitability and preserves the rich cultural diversity. The main benefit relates to securing jobs, refrigeration of food and medicine, purifying and recycling water, and connecting to the world via electronic media.

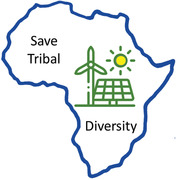

## 1 Introduction

Providing sufficient food has been a continuing challenge for decades in view of the growing human population, in particular in developing countries. Since the beginning of industrialization 250 years ago, i.e., during only 8 out of 10 000 generations that *Homo sapiens* has lived, the world population increased by more than eightfold.^[^ ^1^ ^]^ Meanwhile, the population growth rate is decreasing on all continents so that a flattening out of the population is expected during the current century. While the annual growth rate in Europe has become slightly negative, Africa, with 3.18%, shows by far the highest value, paralleled by the lowest life expectancy and the lowest degree of urbanization.^[^ ^2^ ^]^

In addition to the population increase during industrialization, the use of fossil fuel exploded by a factor of 1400.^[^ ^1^ ^]^ This development had the consequence of far‐ranging social, political, economic and environmental challenges,^[^ ^3^ ^]^ and has led to an increase of atmospheric carbon dioxide (CO_2_) concentration with concomitant temperature rise considerably beyond any levels over the past 800 000 years, as determined from Antarctic ice core analysis.^[^ ^4^, ^5^ ^]^ Considering the fact that people have adapted to live at temperatures between +50 and −50 °C, an increase of the average global temperature by 2 °C as envisaged by the Paris Agreement on Climate Change^[^ ^6^ ^]^ looks negligible. However, by comparison, the preindustrial temperature fluctuations over a period that covered several glacials spanned ca. 8 °C. Therefore, an increase of 2 °C is expected to have severe consequences for life on Earth, with a sea level rise by several meters and changes in habitability and biodiversity due to climatic changes, expanding deserts, and disappearing glaciers.

In recognition of these developments, there are worldwide efforts toward a net‐zero carbon emission economy by replacing fossil fuel with renewable energy, mainly solar photovoltaics, and wind electricity. This implies a fundamental energy transition in industrialized areas of our planet, and the figure of merit is the lowering of CO_2_ emission. However, contributions to the high CO_2_ content of our atmosphere are far from being distributed homogeneously over our planet, rather they are limited mainly to the northern hemisphere, to a large extent to the rich industrialized areas of North America, Europe, and part of Asia, whereas populations in the disadvantaged southern hemisphere, in Africa and South America, are of low income and have contributed rather little to CO_2_ emission.^[^ ^7^ ^]^ The rural areas in particular have not participated in the fierce industrial development and have remained in a pre‐industrial state. These people will also need an energy transition, but climate change is not the prime motivation as in other parts of the world, and CO_2_ emission is thus not a suitable measure of success. Rather than fossil fuel, their energy source is often firewood which counts as renewable since its contribution to CO_2_ emission is recycled by the biosphere so that the carbon footprint is net zero. The necessary transition will skip the fossil fuel area and go directly to renewable electricity that is the preferred form of energy in modern life. Since technological innovations like solar concentrators or renewable energy in general will allow cooking, this will be of particular advantage in regions where firewood is already scarce and where the energy transition will allow environmental conservation by maintaining or even restoring the biosphere. The still rapidly growing population in Africa also means an increased competition for firewood and other biofuels, an effect that will be much less severe when relieved by the energy transition.

We will focus here on rural areas in sub‐Saharan Africa, the home of many of the world's poorest and most vulnerable people where >80% of the population is not connected to an electricity grid.^[^ ^8^ ^]^ According to the United Nations Sustainable Development Goals for Africa,^[^ ^9^ ^]^ access to clean and affordable energy is one of the goals, and it is key to the fundamental goals of good health and well‐being, including life‐saving interventions, quality education and clean water, sanitation, and economic growth.^[^ ^10^ ^]^ We will show how solar and wind energy coupled with electrical storage to alleviate the day–night cycle allow for a significant improvement of living standards in off‐grid locations. Electrical power is needed for water purification, desalination, and recycling, and for operation of refrigerators to preserve medical drugs and keep food fresh. By access to communications and information technology, it permits connecting to the world by television and wireless phone giving access to remote electronic banking, while it nevertheless preserves tribal entity, local culture, language, and religion. Electricity may also contribute to increasing income by facilitating tourism. This will reduce significantly any migratory pressures to larger cities or economically more attractive areas.^[^ ^11^ ^]^ However, while traditional culture will be preserved, the energy transition and its concomitant use of electrical devices imply a different type of cultural transition.^[^ ^12^ ^]^ It is not possible for outsiders to understand and guide the adaptations required for each community and culture in their pursuit of improved living standards. That would indeed negate the intrinsic respect that we should have for cultures different from our own.

The present work is a proposal that addresses government officials, their science advisors, and town planners, highlighting the latest scientific and technological advances in view of developing infrastructure for enhanced health care and general living conditions under conservation of environment and actively supporting the tribal structure and cultural diversity in off‐grid rural areas. It is hoped to prevent the migratory pressure to large cities which often leads to the formation of large squatter camps that do by no means fulfill the hope for better living conditions.

## 2 Results and Discussion

### 2.1 a) Africa is Dark at Night

**Figure 1:**
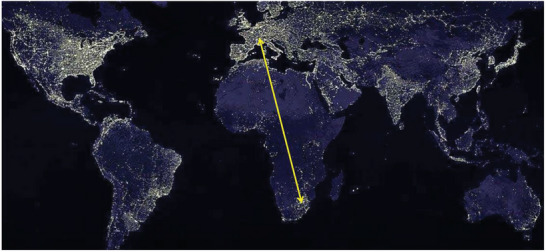
Satellite view of the Earth at night, with yellow arrow showing the flight path between South Africa and Europe (Open source, adapted from ref. [13]).

The dominant fraction of the more than 1.2 billion people on the African continent live without access to an electricity grid. This is impressively seen in a satellite view of our planet at night where Africa is in pronounced contrast not only to the United States and Europe but perhaps surprisingly also to India (**Figure** **1**). No lights are seen on a night‐time flight from Johannesburg to central Europe between Gabarone in Botswana and Algeria. Africa is dark at night, with the exception of its northern rim and the populated area in South Africa.

The absence of access to electricity has severe consequences for the living standard:

- The jobs are where the light is on. Access to electricity will greatly reduce the migratory pressure to areas with better living conditions. Education in specialized subjects without trained teachers, home office and call center jobs are unthinkable without robust satellite internet communication. It is also a prerequisite for small business, eco‐tourism, the employment of volunteers from international organizations, and the potential return of professional expatriates with a wish to return to their roots and serve their communities.
- Health depends to a significant extent on access to potable water. Purification, desalination, and recycling of water require energy. Israel, the world leader in the reuse of water, recycles 80% of its household waste water and uses it mostly for irrigation purposes.^[^ ^14^, ^15^ ^]^ Desalination plants for brackish water and seawater can operate intermittently when energy is available. In addition, they are cost‐efficient and therefore ideal for the use of renewable energy.
- Purification of wastewater or desalination requires the presence of liquid water. However, in arid environments there may not be sufficient liquid water. Recently, an alternative method has been reported that efficiently extracts water from air, even at ≤30% relative humidity. It uses a super‐hygroscopic polymer film that takes up the water which is then desorbed as a liquid by mild heating.^[^ ^16^ ^]^ It is reported to operate for 14–24 cycles per day, which is equivalent to 5.8–13.3 L of water per kg of the polymer.
- Solar‐powered irrigation tools have been proposed to make low‐cost precision agriculture more accessible. Drip irrigation systems release water and nutrients directly to the root zone of the crop through a network of pipes and emitters and can save 20–60% compared to conventional flood irrigation systems.^[^ ^17^ ^]^ The valves are controlled by an App on the mobile phone and on the interference by the farmer.
- Most refrigerators run on electrical energy. Refrigeration is key in keeping food fresh and medical drugs safe. In a continent where the average temperatures are high, this is of particular importance. Modern well‐insulated refrigerators and the latent melting energy of large blocks of ice, match well with the intermittent availability of renewable energy, requiring no (expensive) electrical energy storage.
- Light in a home in the evening is important for family life and learning conditions for students. The recent Covid pandemic has demonstrated how important digital versions of learning are. The ability to connect to the rest of the world by radio, television, and cell phone is standard today in much of the world. It stimulates social and economic progress across a country. There appears to be a clear correlation between broadband availability and jobs and Gross Domestic Product growth.^[^ ^18^ ^]^ The cell phone even brings the all‐important access to the bank to remote villages.
- Access to reliable electricity can help provide the infrastructure, education, and health services deemed essential to ensure that diversity does not come at the cost of societal cohesiveness. A highly fractionalized Sub‐Saharan Africa has indeed been identified as a growth stumbling block. Economic productivity, measured by the logarithm of the night light intensity (Figure 1) shows a “hump‐shaped” effect on population homogeneity, reflecting a trade‐off between the beneficial and the detrimental effects of diversity on productivity.^[^ ^19^ ^]^

### 2.2 b) Africa's Rich Cultural and Societal Diversity

**Figure 2:**
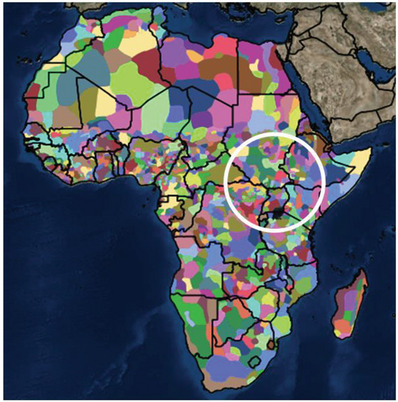
Africa's rich and precious ethnic diversity, overlaid with country borders. The white circle represents the commonly assumed origin of *Homo sapiens* (adapted from National Geographic, open access blog.^[^ ^21^ ^]^).

The African continent represents a very rich cultural diversity with unique tribal identities, native languages, religious beliefs, rituals, rites, dances, dishes, arts and crafts, greetings, and other traditions, even varying laws and morals. The diversity patches survived the colonial era and often change within short distances or even from one village to the next (**Figure** **2**). With the exception of the city of Cairo and the Johannesburg–Pretoria area this is particularly evident in the fertile belt around the equator where the rural population density is highest. Naturally, cultural entities take up more space in the low‐populated Saharan area and in the Kalahari Desert adjacent to the equatorial belt. The original ancestors of *Homo sapiens*, some 300 000 years ago, are believed to have lived in the eastern part of the African equatorial belt from where migration occurred to other parts of the world. According to Galor,^[^ ^20^ ^]^ it is not a coincidence that diversity is highest at its origin and depletes as a function of distance along the migratory path because not all cultural groups participate to the same extent in migration.

There is no question that diversity deserves to be preserved as much as possible, social diversity must be respected as we do bio‐diversity for the sake of long‐term human survival. However, unchecked migration from rural areas to big cities or other countries will inevitably lead to their mixing and loss. Improved living conditions will allow people to remain in their traditional environment and benefit from their family and tribal contacts. This will protect and preserve their culture, language, and religion. People need a realistic choice about where and how they want to live. Access to electricity is therefore a deciding feature that allows them to build their own life in a Future Africa.

The downside of diversity is that it can also be seen as an obstacle against unity,^[^ ^19^ ^]^ Africa has currently 54 sovereign states, and it is unfortunately true that in particular governments of centralized states are often reluctant to allow different provinces or even smaller units to adhere to different values because it tends to lead to separatist activities to achieve independence. Indeed, culture can also act as an impediment and complicate attempts at promoting more efficient, more sustainable, and often more affordable use of mobility as well as energy use in homes and buildings.^[^ ^12^ ^]^ The success of developments will depend on the wisdom of governments, i.e., how they allow minorities to be represented in political bodies and in television programs, e.g. in terms of local languages, and how much decision‐making is decentralized and deferred to provinces and tribal communities. Diversity may increase the incidence of civil conflicts, thus adversely affecting economic performance. On the other hand, it may stimulate cross‐fertilization of ideas in innovative activities, facilitating rapid adaption to changing technological environments.^[^ ^19^ ^]^

India represents a partly contrasting case to Africa. With its currently spoken 780 languages it shows a high diversity akin to Figure 2. However, Figure 1 gives a different view, as India is not dark at night. Much of its area is connected to a national electricity grid, although with limited reliability. Furthermore, 400 out of the 780 languages are at risk of extinction in the next 50 years.^[^ ^22^ ^]^ This demonstrates the danger for diversity in a highly integrated and centrally organized nation. The availability of renewable energy supports decentralized structures and could perhaps also reduce the pressure regarding enhanced political centralization.

A different aspect is given by the fact that an open fire plays a significant role for families as a space to gather and eat and talk.^[^ ^12^ ^]^ Solar panels may reduce the use of firewood and save the trees, but new traditions to establish family life have to be developed when the open fire disappears. Furthermore, if firewood is no longer used, there will be no smoke to cure pre‐salted fish or meat; instead, people will have to get used to keep food in a freezer.^[^ ^12^ ^]^ One could, however, also argue that alternative energy sources, e.g., for hot water, cooking, and washing, could reduce wood use to sustainable levels to still allow for important traditional uses of fire like for social gatherings or meat curing.

Galor, in his book “The Journey of Humanity”,^[^ ^20^ ^]^ argues what happened when *Homo sapiens* left Africa is analogous to the reduction of diversity when a colorful parrot population is blown away by a hurricane and dispersed from an island. This pattern was repeated with each onward migration. Africa is the most diverse place on the planet, genomically and culturally, and diversity has a knock‐on effect on prosperity. It accounts for about a quarter of the otherwise unexplained variation between nations, Galor calculates; in contrast, diseases (the Tsetse fly, malaria, etc.) account for one‐seventh, and political institutions (democracies vs autocracies) less than one‐tenth. What is it about diversity that makes such a big impact? Social cohesiveness – low diversity, in other words – can have its benefits, particularly in earlier phases of development. But in the modern world, or the boiling kettle phase, cultural fluidity is the greatest driver of innovation. “Like biological breeding, the mating of ideas … benefits from a broader pool of individuals,” he writes. That mating of ideas gives rise to new policies, new inventions, and enhanced productivity, stoking the engine of growth. Culturally fluid societies are more likely to be able to adapt to changing conditions.

### 2.3 c) The Technical Approach to Off‐Grid Habitability

It is useful here to consider some of the technical implications for an off‐grid energy transition with first‐time use of electricity.

The worldwide commitment to abandon the use of fossil fuels leads to decentralized production of renewable energy, which perfectly matches off‐grid demands. The most basic solution consists of solar hot water generation and concentrators delivering heat for cooking. Much more versatility is obtained with the availability of electricity, driven by solar photovoltaic panels and wind turbines which can be installed locally. The preference for one or the other method is based on the local climatic conditions. However, while solar irradiation exhibits strong day–night cycles, there is some complementarity between the two options in the sense that wind blows also in bad weather and at night, in fact, the windiest conditions are often found after sunset when the demand for electrical power in private homes is at its maximum. Therefore, for installations that serve the population of an entire village it is advisable to utilize both solar and wind energy.

Both these methods have been available for several decades, so that their use is routine. The required skills to install and service these technologies are modest and can be managed by people with minimum training. An interesting and successful solution to manage this energy transition in rural areas has been developed based on private initiative by Bunker Roy in India who founded the Barefoot College.^[^ ^23^, ^24^ ^]^ This institution provides practical training to mostly illiterate women and prepares them to install and maintain solar panels and solar concentrators back in their home villages. It would be wise to follow this example and initiate regional training centers in Africa that can provide this training and help with attracting funding. It is of particular relevance that societies without a tradition of incorporating technical devices need to develop a culture of maintenance and repair in order to keep new installations functional over many years.^[^ ^12^ ^]^

To compensate for the cyclic availability of these resources, it is recommended to amend the installations by battery storage. The full cost of solar and wind electricity (not counting storage) has dropped below the price of electricity from the grid (assuming 0.05 US$ per kWh) so this is also a competitive economic option. Nevertheless, engagement and advice by communities and governmental bodies, backed by funding from international institutions, are required to enter into small off‐grid energy systems.

Basic electric energy needs such as lighting and small appliances in a five‐person household are initially in the range of 34–420 kWh per year and expected to increase toward 1800 kWh per year after electrification.^[^ ^8^ ^]^ A 1 m^2^ photovoltaic panel is expected to provide 200 kWh per year under standard test conditions (1000 W m^−2^ solar irradiance for 2.74 h a day). These numbers may serve only as a rough guide since they vary considerably over the year and depend on geographic latitude on the continent due to the local weather conditions. To cope with day‐night cycles and with bad weather periods it is advisable to install complementary storage with a capacity of several days. For comparison, today's lithium‐ion (Li‐ion) batteries have a storage capacity on the order of 0.25 kWh kg^−1^, so that such a battery of 10–20 kg would cope with the typical storage requirements for several days. Planning for uninterrupted refrigeration is crucial.

Although the present state‐of‐the‐art Li batteries like the Tesla Powerwall with a storage capacity of 13.5 kWh at 120 kg weight perform excellently, an attractive alternative for stationary applications is the saltwater battery. It is an intercalation battery similar to the Li‐ion battery, has ≈50% of the Li‐ion energy density and half of its cycling rate, but it has the important advantages that it is much more robust, in particular as regards the large operating temperature range (−20 to +60 °C), that it can be completely discharged without any damage, is completely maintenance‐free, its raw materials (aqueous sodium sulfate solution as electrolyte, intercalating titanium phosphate anode and magnesium oxide cathode, synthetic fleece separator, stainless steel current collector and housing) are non‐toxic and abundantly available around the world. It is non‐corrosive, can neither burn nor explode, and is thus considered absolutely safe. Its lifetime is 15+ years (>5000 cycles), after which it can be recycled. Although the technology is still young, this battery looks highly suitable for solar energy storage in rural off‐grid applications.^[^ ^25^ ^]^

Other types of batteries that are available for stationary electricity storage under off‐grid conditions include used lead‐acid batteries from trucks, various kinds of flow batteries, and the emerging robust, low‐toxicity, and long‐lived solid electrolyte batteries.^[^ ^26^ ^]^

An important requirement for habitability is the availability of clean and sterile and thus potable water. First, it is important to avoid using waste water from mining applications as these will often be contaminated by toxic metal ions.^[^ ^27^, ^28^ ^]^ A simple method to disinfect water when cookers are unavailable uses transparent glass or intact polyethylene terephthalate (PET) beverage bottles which are filled with clear or filtered water and deposited in full sunlight for at least 6 h with occasional shaking. The state‐of‐the art technology for wastewater recycling and also for desalination of seawater is reverse osmosis. This is an energy‐intensive process, in particular in the case of desalination which has to operate at much higher pressures across the semipermeable membrane. A number of low pressure‐drop membranes are, however, presently being researched. Wind energy, photovoltaic, or solar thermal energy are useful in small or spatially confined communities, more so than for big cities.^[^ ^29^ ^]^ Thus, renewable energy enables to follow the principle of reducing, reusing, and recycling water, but one should be aware that the availability of pure water also catalyzes population growth.^[^ ^30^ ^]^ This argument certainly does not hold for communities that offer – especially to females – an opportunity of formal schooling till the age of 16 years and children are relieved of traditional tasks such as carrying firewood and water.

It is appropriate to say a few words about the type of electricity networks to be used. Long‐distance transfer of electricity is at high voltage because this reduces proportionally the waste heat from Ohmic resistive heating in the leads. For local grids, with distances below a couple of kilometers, this is less important. Second, the final distribution is in most cases as alternating current (AC) electricity down to the outlets in our homes. This is mostly for historical reasons, because AC was much easier to transform to high voltages than direct current (DC). Today, transforming DC current is easier, even allowing long‐distance high voltage, low‐loss DC power lines between different countries.

Photovoltaic (PV) modules always produce low‐voltage DC electricity, and connecting different cells in series allows adding up the voltages of the individual cells. Alternatively, using solar power inverters, it can be converted to AC for a conventional grid and home appliances. Wind turbines can produce DC or AC power depending on the type of generator used. On the consumer side, all electronic devices like cell phones, laptops, flat‐screen television sets, light emitting diode (LED) lights, and hybrid and fully electric vehicles, operate with low‐voltage DC, reflecting the band gaps of a few eV of typical semiconductors. Some of these, like TVs and certain LED light bulbs, use an internal converter, others use an external one to reduce weight for carrying the device around. Most motors run on AC but can also be built to use DC, with each mode having its advantages and disadvantages. Solar‐powered DC refrigerators are commercially available with a separate external converter to be used with a standard household AC plug. Inductive hotplate heating requires AC power, whereas resistive heating does not distinguish between AC and DC.

The local availability of renewable energy in large and strongly cyclic quantities is a challenge for available AC electricity grids. Local storage in batteries is in DC form, and since consumer electronics also require low voltage DC power, the installation of DC mini‐grids should be evaluated, especially in new, first‐time electrical installations, that avoid the losses in repeated transformation between AC and DC that can amount to as much as 20%. Furthermore, as a considerable advantage, the batteries included in DC grids provide automatic stabilization without external energy management.

It is pleasing to see that electrification activities based on solar mini‐grids are already underway at selected places in Africa, notably in Nigeria, with plans to expand to other sub‐Saharan countries. These efforts involve significant financial investments supported by the World Bank and private investors.^[^ ^31^ ^]^

Although we have emphasized renewable energy and satellite communication as pillars for off‐grid sustainable development, it should be mentioned that most of the United Nations Sustainable Development Goals can in future be addressed by technology that is presently being researched to improve the carbon footprint of modern living. Examples are the exciting developments in low‐carbon cement production, bio‐materials for construction and clothing (wood, bamboo, grass), recycling urine, and other fertilizer manufacturing processes that bypass the energy‐intensive Haber–Bosch synthesis of ammonia, decentralized sewerage systems and the production of biogas from dung and other organic material, no‐till, and water efficient farming methods. All of these technologies are down‐scalable to small villages. Electric transport suitable for off‐grid communities including electric off‐road motorcycles and scooters designed for hauling produce have already become commercially available.

Nexgen, the new administrative capital near Cairo, will be the first climate‐positive metropolis in the world.^[^ ^30^ ^]^ Another futuristic solar, wind, and hydrogen energy‐based gigacity that is designed from scratch and currently under construction is NEOM in Saudi Arabia.^[^ ^32^ ^]^ These examples demonstrate that it is much better to start from scratch than to fix up the present monster cities of the world! However, this top‐down approach creates a massive cultural melting pot. It is not suitable to preserve diversity and is in pronounced contrast to the cautious bottom‐up approach that we are suggesting for the villages in off‐grid rural areas.

Economics of scale and international trade in massive items like ores, commodities, staple foods, motor cars, is what led to our present climate dilemma. If we only trade in small items like chips, PV cells, and information, we should do fine without mass transport. The renewed interest in off‐grid sustainable living is a fascinating scientific and economic challenge.

## 3 Conclusion and Recommendations

Access to energy, in particular to electricity, is a fundamental pillar of today's living standard. Off‐grid locations will skip the stage of extensive use of fossil fuels and enter the age of renewable energy directly. Wind and solar energy are ideally suited for local solutions without having to build an expensive electricity grid. These technologies have matured and they are more than competitive with coal as regards price of electricity. Their cyclic availability requires transient storage. Batteries have made enormous progress for direct short‐term electricity storage. This obviates indirect storage via transformation to chemical forms like hydrogen or synthetic solar fuels which are still a viable solution for long‐term storage of larger amounts of energy.^[^ ^33^ ^]^

Access to electricity will be essential for human health and well‐being as it allows refrigeration of food and medication, drinking water purification, and connection to an increasingly digital world via television and internet. This will prevent migration from rural areas to the large cities and thus contribute to the preservation of the rich cultural diversity across the African continent. Nevertheless, even if there is success in preventing migration to the cities, access to electricity and electronic media also implies a cultural transition and social adaption, but it will be much smoother and less disruptive than dislocation into a new environment with culturally mixed, urban profile.

Due to the large African annual population increase of 3.2% but also adaption to increasing living standard as a consequence of industrialization, the energy demand is expected to increase significantly.^[^ ^33^ ^]^

The energy transition to renewable electricity will not be automatic, since, due to the lack of media, information is still scarce in rural areas, and the financial possibilities are also limited. This calls for initiatives by the government or by developmental organizations. The private Indian initiative discussed above, the Barefoot College,^[^ ^23^, ^24^ ^]^ is a promising model because it teaches local people for setting up and maintaining photovoltaic equipment. This has to be combined with governmental subsidy, microcredits or support by international developmental programs. Thereby, organic growth is preferable since it is less disruptive and likely less susceptible to corruption than a rapid large area‐dictated overthrow of current economic and social order.

The underdeveloped part of Africa is not responsible for climate change but could use the fascinating science and technology designed for this international challenge to develop an off‐grid sustainable model for addressing physical well‐being and the preservation of a threatened, irreplaceable cultural diversity. An unforeseen opportunity for off‐grid development has arisen from the crisis‐need to develop a range of novel low‐carbon technologies that inevitably are down‐scalable, as exemplified by the photo‐voltaic cell.

## Conflict of Interest

The authors declare no conflict of interest.

## References

[1] W. E. Rees , World 2023, 4, 509.

[2] PopulationStat , World Statistical Data, populationstat.com/continents (accessed: October 2023).

[3] S. Asem ‐ Hiablie , D. D. Uyeh , A. Adelaja , K. Gebremedhin , A. Srivastava , K. Ileleji , M. Gitau , Y. Ha , T. Park , Global Chall. 2023, 7, 2300033. 10.1002/gch2.202300033 PMC10517289 37745824

[4] E. Brook , Nature 2008, 453, 291. 18480800 10.1038/453291a

[5] D. Lüthi , M. Le Floch , B. Bereiter , T. Blunier , J.‐M. Barnola , U. Siegenthaler , D. Raynaud , J. Jouzel , H. Fischer , K. Kawamura , T. F. Stocker , Nature 2008, 452, 379. 10.1038/nature06949 18480821

[6] United Nations , Climate Change, unfccc.int/process‐and‐meetings/the‐paris‐agreement/d2hhdC1pcy (accessed: October 2023).

[7] H. Winkler , South African J. Sci. 2018, 114, 17.

[8] R. Muhumuza , A. Zacharopoulos , J. D. Mondol , M. Smyth , A. Pugsley , Renew. Sustain. Energy Rev. 2018, 97, 90.

[9] United Nations , Department of Economic Affairs, Sustainable Development, sdgs.un.org/goals (accessed: October 2023).

[10] A. Franco , M. Shaker , D. Kalubi , S. Hostettler , Sust. Energ. Technol. Assessments 2017, 22, 92.

[11] E. Roduner , E. R. Rohwer , in Environmental Humanities of Extraction in Africa (Eds: J. Ogude , T. Mushonga ), Taylor and Francis, London, 2022, Ch. 11.

[12] B. K. Sovacool , S. Griffiths , Renew. Sustain. Energy Rev. 2020, 119, 109569. 10.1016/j.rser.2021.110919 PMC9183457 35702384

[13] NASA NOAA , Earth at Night, www.nasa.gov/image‐article/earth‐night (accessed: October 2023).

[14] National Water Reuse Action Plan , Lessons from Israel's water reuse approach, www.epa.gov/system/files/documents/2023‐03/From%20Water%20Stressed%20to%20Water%20Secure%20‐%20Lessons%20from%20Israel%27s%20Water%20Reuse%20Approach.pdf (accessed: October 2023).

[15] T. Richards , J. H. Harrhy , R. J. Lewis , A. G. R. Howe , G. M. Suldecki , A. Folli , D. J. Morgan , T. E. Davies , E. J. Loveridge , D. A. Crole , J. K. Edwards , P. Gaskin , C. J. Kiely , Q. He , D. M. Murphy , J.‐Y. Maillard , S. J. Freakley , G. J. Hutchings , Nat. Catal. 2012, 4, 575.

[16] Y. Guo , W. Guan , C. Lei , H. Lu , W. Shi , G. Yu , Nat. Commun. 2022, 13, 2761. 35589809 10.1038/s41467-022-30505-2 PMC9120194

[17] A. Wilson , MIT News, Smart irrigation technology covers “more crop per drop”, news.mit.edu/2023/gear‐lab‐creates‐affordable‐user‐driven‐smart‐irrigation‐controller‐1025 (accessed: October 2023).

[18] P. Lundmark , Smart Cities are Great but We Also Need Smart Villages, The European Sting, Brussels, Belgium, 11 October 2021.

[19] T. S. Mashau , L. R. Kone , H. N. Mutshaeni , J. Sociol. Soc. Anthropol. 2015, 6, 235.

[20] O. Galor , The Journey of Humanity – The Origin of Wealth and Inequity, Random House, UK 2022, p. 9781847926913.

[21] National Geographic , Education Blog, blog.education.nationalgeographic.org/2015/02/18/africas‐dazzling‐diversity (accessed: October 2023).

[22] S. Santoshini , Centering Indigenous Languages in India's Schools, www.yesmagazine.org/social‐justice/2023/10/23/india‐schools‐indigenous‐languages‐education (accessed: October 2023).

[23] Barefoot College International, Program, www.barefootcollege.org/solution/solar (accessed: October 2023).

[24] R. Bunker , Learning from a Barefoot Movement, www.ted.com/talks/bunker_roy (accessed: October 2023).

[25] dynamicslr, All you need to know about saltwater batteries, www.bluesky‐energy.eu/en/saltwater_battery, www.dynamicslr.com/all‐you‐need‐to‐know‐about‐saltwater‐batteries (accessed: October 2023).

[26] D. H. S. Tan , Y.‐T. Chen , H. Yang , W. Bao , B. Sreenarayanan , J.‐M. Doux , W. Li , B. Lu , S.‐Y. Ham , B. Sayahpour , J. Scharf , E. A. Wu , G. Deysher , H. E. Han , H. J. Hah , H. Jeong , J. B. Lee , Z. Chen , Y. S. Meng , Science 2021, 373, 1494. 34554780 10.1126/science.abg7217

[27] W. Utembe , E. M. Faustman , P. Matatiele , M. Gulumian , Hum. Exp. Toxicol. 2015, 34, 1212. 26614808 10.1177/0960327115600370

[28] B. Van der Bruggen , Nat. Rev. Chem. 2021, 5, 217. 37117286 10.1038/s41570-021-00264-7

[29] E. Roduner , E. R. Rohwer , Clean Technol. Environ. Policy 2021, 23, 475.

[30] L. Cowan , First Climate‐positive, net zero city in the World, InHabitat , https://inhabitat.com/first-climate-positive-net-zero-city-in-the-world/ (accessed: May 2022).

[31] T. Kene‐Okafor , New solar mini‐grids in Africa powered by Husk Power System’ $103 M Series D, techcrunch.com/2023/10/24/new‐solar‐mini‐grids‐in‐africa‐to‐be‐powered‐by‐husk‐power‐systems‐103m‐series‐d (accessed: October 2023).

[32] M. M. G. Alkeid , Degree project KTH Stockholm, www.diva‐portal.org/smash/get/diva2:1251927/FULLTEXT01.pdf (awarded: September 2018).

[33] G. Mutezo , J. Mulopo , Renew. Sust. Energy Rev. 2021, 137, 110609.

